# Exploring the Potential of Metal–Organic Frameworks for the Separation of Blends of Fluorinated Gases with High Global Warming Potential

**DOI:** 10.1002/gch2.202200107

**Published:** 2022-10-21

**Authors:** Julio E. Sosa, Carine Malheiro, Paulo J. Castro, Rui P. P. L. Ribeiro, Manuel M. Piñeiro, Frédéric Plantier, José P. B. Mota, João M. M. Araújo, Ana B. Pereiro

**Affiliations:** ^1^ Department of Chemistry LAQV REQUIMTE NOVA School of Science and Technology NOVA University Lisbon Caparica 2829‐516 Portugal; ^2^ Université de Pau et des Pays de l'Adour E2S UPPA CNRS TOTAL LFCR Anglet 64600 France; ^3^ Departamento de Física Aplicada Facultade de Ciencias Universidade de Vigo Vigo E36310 Spain

**Keywords:** F‐gases, gas separation, HFCs, porous materials, refrigerants

## Abstract

The research on porous materials for the selective capture of fluorinated gases (F‐gases) is key to reduce their emissions. Here, the adsorption of difluoromethane (R‐32), pentafluoroethane (R‐125), and 1,1,1,2‐tetrafluoroethane (R‐134a) is studied in four metal–organic frameworks (MOFs: Cu‐benzene‐1,3,5‐tricarboxylate, zeolitic imidazolate framework‐8, MOF‐177, and MIL‐53(Al)) and in one zeolite (ZSM‐5) with the aim to develop technologies for the efficient capture and separation of high global warming potential blends containing these gases. Single‐component sorption equilibria of the pure gases are measured at three temperatures (283.15, 303.15, and 323.15 K) by gravimetry and correlated using the Tóth and Virial adsorption models, and selectivities toward R‐410A and R‐407F are determined by ideal adsorption solution theory. While at lower pressures, R‐125 and R‐134a are preferentially adsorbed in all materials, at higher pressures there is no selectivity, or it is shifted toward the adsorption R‐32. Furthermore, at high pressures, MOF‐177 shows the highest adsorption capacity for the three F‐gases. The results presented here show that the utilization of MOFs, as tailored made materials, is promising for the development of new approaches for the selective capture of F‐gases and for the separation of blends of these gases, which are used in commercial refrigeration.

## Graphical abstract

Four different metal–organic frameworks (MOFs) and one zeolite are studied for the adsorption of fluorinated gases and are evaluated for the separation of refrigerants with high global warming potential. The utilization of MOFs for these purposes is promising and by selecting materials with different porous characteristics and chemistries, it is possible to fine‐tune the separation of commercial refrigerants.

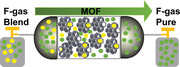

## 1 Introduction

Climate change and global warming, resulting from human actions, are endangering the sustainability of the planet and human life. Therefore, the reduction of the emissions of compounds that deplete the ozone layer and/or have high global warming potential (GWP) has been a top priority in the last decades. Humanmade fluorinated greenhouse gases (F‐gases), such as hydrofluorocarbons (HFCs), have been essential to human life, being used in a large range of industrial applications, such as air conditioning and refrigeration systems.^[^ ^1^ ^]^ These gases started to be massively used after the Montreal Protocol because they are energetically efficient, do not damage the ozone layer, and have low levels of toxicity and flammability.^[^ ^2^ ^]^ However, they are powerful greenhouse gases with a GWP up to 23 000 times greater than CO_2_ and with long atmospheric lifetimes.^[^ ^1^, ^2^, ^3^ ^]^ Then, international agreements were signed aiming at reducing substantially the emissions of these gases, such as the Kigali international agreement which was signed in 2016.^[^ ^4^ ^]^ Moreover, in the European Union the 2014 F‐gas legislation targets to cut emissions by two‐thirds by 2030.^[^ ^5^ ^]^ Thus, the research on green and sustainable technologies to efficiently capture, separate, and recycle F‐gases is a top priority to accomplish the climate goals and to make the refrigeration and air conditioning sector more sustainable.

In the last decades, the market has been dominated by the so‐called third‐generation refrigerants, which include R‐134a (1,1,1,2‐tetrafluoroethane) and blends, such as R‐407F and R‐410A, containing R‐32 (difluoromethane), R‐125 (pentafluoroethane), and R‐134a, among others HFCs. However, most of these refrigerants have been banned or are being phased out, and a transition to fourth‐generation refrigerants with lower GWP is currently underway.^[^ ^6^, ^7^ ^]^ These fourth‐generation refrigerants include R‐444A, R‐447A, and R‐448A, which are blends of HFCs (R‐32, R‐125, R‐134a, and others) with hydrofluoroolefins (HFOs).

Despite the imposed legislation that controls the introduction of new high GWP refrigerants in the market, a great amount of these refrigerants is still present in much of the equipment used nowadays. Therefore, the correct management of the gases recovered from end‐of‐life equipment will be vital in the next decades. Considering the phase‐out of third‐generation refrigerants and the possible utilization of the components of these refrigerants to produce low‐GWP fourth‐generation refrigerants, the recovery and separation of HFCs (such as R‐32, R‐125, and R‐134a) from HFC blends (such as R‐410A and R‐407F) is of vital importance. It will allow not only to avoid the release of these gases into the atmosphere but also to recycle them and produce blends with HFOs (fourth‐generation refrigerants), applying a circular economy which is a priority in Europe. However, several blends used as refrigerants have azeotropic or near‐azeotropic behavior and therefore are not easily separated by conventional methods, such as distillation. In the last years, the separation of F‐gas blends through low‐cost and energy‐efficient adsorption and membrane technologies has been explored, with promising results.^[^ ^8^, ^9^, ^10^, ^11^ ^]^

The adsorption of these gases on activated carbons, zeolites, or metal–organic frameworks (MOFs), as well as in alternative solvents such as ionic liquids, at various temperature and pressure ranges has been reported in the literature.^[^ ^9^, ^12^, ^13^, ^14^, ^15^ ^]^ In the last decades, the investigation of porous materials for separation processes has been greatly advanced. The utilization of materials with different properties, such as surface area and pore volume and dimensions, and functionalization allow their selectivity toward a specific species of a gas blend, allowing to separate it from the other components of the mixture. Zeolites are microporous materials, typically with pore size in the range of <2 nm and surface area in the range of <1000 m^2^ g^−1^.^[^ ^14^, ^16^, ^17^ ^]^ In contrast, both activated carbons and MOFs can be micro‐mesoporous materials, with pores size in the range of 2–50 nm, and activated carbons may also have macropores (>50 nm).^[^ ^14^, ^18^, ^19^ ^]^ These materials also have significant differences relative to their surface areas, with activated carbons having surface areas of over 1000 m^2^ g^−1^ and MOFs having surface areas of up to 10 000 m^2^ g^−1^.^[^ ^14^, ^18^, ^19^ ^]^ Thus, according to the type of gas for which an adsorption or separation process is intended to be designed, different types of adsorbents can be selected.

Zeolites have been studied for the capture and separation of F‐gases mixtures, and strong interactions have been identified between zeolites and HFCs with high reported heats of adsorption.^[^ ^11^ ^]^ Zeolites 4A, 5A, and 13X have been studied for the separation of refrigerants from unwanted by‐products (e.g., separation of R‐134/R134a and R‐23/R‐22 mixtures) and of refrigerants composed of binary and ternary mixtures of R‐22, R‐32, R‐125, R‐134a, and R‐143a.^[^ ^20^, ^21^, ^22^, ^23^, ^24^, ^25^, ^26^ ^]^ ZSM‐5 is a manufactured zeolite with a regular channel network of pore aperture around 5–6 Å, showing high thermal stability and a surface topology that makes it advantageous for catalysis and sorption. It has been studied for pure and multicomponent adsorption of different gases, such as CO_2_, N_2_, methane, ethylene, and propane,^[^ ^27^, ^28^, ^29^, ^30^ ^]^ but no reports of HFC sorption are available in the literature.

MOFs have a highly porous structure, high chemical and thermal stability, and high tunability, with a great variety of possible chemical compositions, and different shapes of building units and architectures. These characteristics make them optimal candidates for several applications such as capture and storage of gas molecules and large molecules, and catalysis.^[^ ^19^, ^31^ ^]^ However, the application of these materials in HFCs separation processes is still quite unexplored.^[^ ^32^ ^]^ In fact, only a few studies have been conducted to study the capture and/or separation of HFCs, such as R‐32,^[^ ^33^, ^34^, ^35^ ^]^ R‐125,^[^ ^33^ ^]^ and R‐134a.^[^ ^33^, ^35^, ^36^ ^]^ The available data include studies with Zr‐UiO‐66, MOF‐5, Mg‐MOF‐74, Ni‐MOF‐74, Co‐DOBDC, Ni‐DOBDC, Fe‐MIL‐100, Cr‐MIL‐101, and Cu‐benzene‐1,3,5‐tricarboxylate (BTC).^[^ ^33^, ^34^, ^35^, ^36^ ^]^ However, a huge variety of subclasses of MOFs with different topologies and properties can be used for F‐gas capture and separation processes. Cu‐BTC (also known as HKUST‐1 or MOF‐199) has a framework of dimeric Cu^2+^ metal centers coordinated with oxygen atoms from BTC linker molecules. It is one of the most well‐characterized MOFs, with a relatively easy synthesis and high thermal stability, and it has been studied for a variety of applications, including catalysis and gas capture and separation.^[^ ^37^ ^]^ It has been widely studied for the capture of a variety of gases, including H_2_, CO_2_ and SF_6_, and for the separation of gas mixtures, such as CO_2_/N_2_ and R‐32/R‐125.^[^ ^33^, ^38^ ^]^ The MIL‐53 class is characterized by containing a metal center (typically a trivalent one, such as Al^3+^) and terephthalate (benzene‐1,4‐dicarboxylate) as linker molecule, presenting structural flexibility that results in a transition between a narrow‐pore and a large‐pore conformation, triggered by the adsorption of specific molecules. In recent years, this class of MOFs has been evaluated for the adsorption of gases, such as carbon dioxide, methane, and hydrogen sulfide.^[^ ^39^, ^40^ ^]^ MIL‐53 (Al) is extremely stable at high temperatures.^[^ ^36^ ^]^ MOF‐177 consists of Zn_4_O tetrahedrons connected with benzene tribenzoate ligands and has been studied, with promising results, for H_2_ adsorption. It presents an exceptionally high specific surface area and large pore volumes. Moreover, it has been also studied for the adsorption of other gases, such as CO_2_, CO, CH_4_, and N_2_O.^[^ ^41^, ^42^ ^]^ Zeolitic imidazolate framework (ZIF) is a subclass of MOFs with a sodalite topology and is composed of imidazolate linkers and metal ions, with structures similar to zeolites. They have tunable pore sizes and can be used in various applications such as chemical sensors, optical switches, biomedical applications, catalysis, as well as gas capture and separation.^[^ ^43^ ^]^ ZIF‐8, a zinc‐based ZIF, has been studied for the selective adsorption of GHGs, including CO_2_.^[^ ^44^ ^]^

In this work, the commercial refrigerants R‐410A (R‐32/R‐125 blend) and R‐407F (R‐32/R‐125/R‐134a blend) were chosen as case studies for the evaluation of advanced materials for the selective separation of value‐added pure HFC from refrigerants with high GWP. Furthermore, we aimed at stepping forward in the study of MOFs for the selective capture of the HCFs R‐32, R‐125, and R‐134a, in order to develop effective processes for the separation of these gases from gas blends used in refrigeration. Four MOFs with different morphologies and textural properties (Cu‐BTC, ZIF‐8, MOF‐177, and MIL‐53(Al)) were selected to gain insights into the MOFs characteristics that affect the interactions with HFCs. Moreover, this study was complemented with one zeolite, ZSM‐5, for which no data are available in the literature on the adsorption of HFCs. Taking into account the good results obtained so far with zeolites, the comparison of both materials will allow us to evaluate whether MOFs improve the separation power of F‐gases from commercial refrigerants.

## 2 Results and Discussion

### 2.1 Materials Characterization

**Figure 1:**
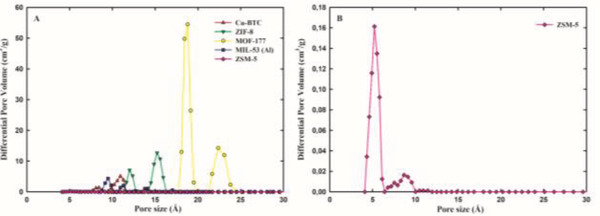
Pore size distribution of the studied adsorbents, determined by deconvolution of the N_2_ (77 K) or Ar (87 K) adsorption isotherms using standard methods based on the nonlocalized density functional theory (NLDFT). Panel A: Cu‐BTC (red up triangles), ZIF‐8 (green down triangles), MOF‐177 (yellow circles), MIL‐53 (Al) (blue squares), and Zeolite ZSM‐5 (pink diamond). Panel B: Zeolite ZSM‐5 (pink diamond).

F‐gas adsorption was studied on four MOFs and one zeolite (ZSM‐5) with different textural characteristics. The specific Brunauer–Emmett–Teller (BET) surface area, *S*_BET_, and specific pore volume, *v*_p_, of each adsorbent are listed in **Table** **1**. The pore size distributions (PSDs) for each material are depicted in **Figure** **1**.

**Table 1:** BET surface area, *S*_BET_, and specific pore volume, *v*_p_, of the zeolite and MOFs

	Cu‐BTC	ZIF‐8	MOF‐177	MIL‐53(Al)	ZSM‐5
*S*_BET_ [m^2^ g^−1^]	1172	1302	4190	1043	434
*v*_p_ [cm^3^ g^−1^]	0.56	0.65	1.75	0.79	0.26

Gas sorption in porous materials is governed by different processes, according to the operating conditions and the characteristics of both the adsorbate (sub‐ or supercritical adsorption) and the adsorbent. Adsorption in microporous materials takes place in the porous network of the adsorbent and therefore is dependent on its textural properties, morphology, and pore size distribution. MOF‐177 is the adsorbent with the highest value of both *S*_BET_ and *v*_p_ (4190 m^2^ g^−1^ and 1.75 cm^3^ g^−1^, respectively), substantially different from the values for the other MOFs (*S*_BET_ values ranging from 1043 to 1303 m^2^ g^−1^, and *v*_p_ values ranging from 0.56 to 0.79 cm^3^ g^−1^) and from the zeolite, which present the lowest values for all materials (*S*_BET_ of 434 m^2^ g^−1^ and *v*_p_ of 0.26 cm^3^ g^−1^).

All materials are clearly distinguishable by their PSD profiles (Figure 1). The PSDs of all materials present two main peaks. While MOF‐177 has both micropores (pores size 17–20 Å) and mesopores (pores size 20–25 Å), zeolite ZSM‐5 (pores size range: 5–10 Å), MOFs Cu‐BTC (pores size range: 7–12 Å), MIL‐53 (Al) (pores size range: 8–10 Å), and ZIF‐8 (pores size range: 12–17 Å) only have micropores (pore size <20 Å). In the case of ZSM‐5, a large fraction of the pores is in the ultramicropore region (bellow 5 Å). These differences in textural properties, together with the differences in the chemical properties of the adsorbents, impact gas adsorption and separation because the physical and chemical processes governing adsorption in mesopores are different from those in micropores.

### 2.2 Adsorption Equilibrium of R‐32, R‐125, and R‐134a

**Figure 2:**
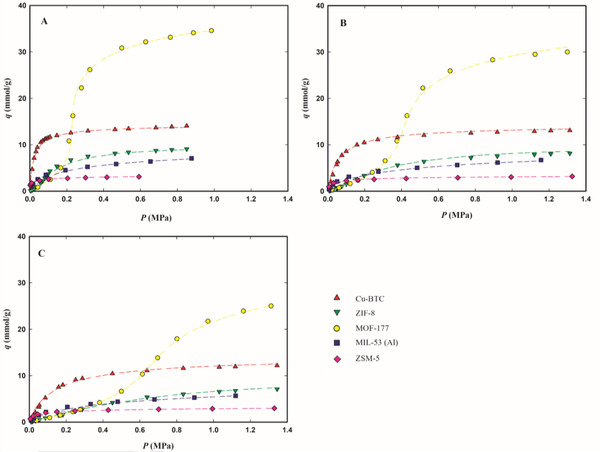
Adsorption equilibrium isotherms of R‐32 on Cu‐BTC (red up triangles), ZIF‐8 (green down triangles), MOF‐177 (yellow circles), MIL‐53(Al) (blue squares), and zeolite ZSM‐5 (pink diamond), at 283.15 K (panel A), 303.15 K (panel B), and 323.15 K (panel C). The dashed lines represent the data fittings with the Tóth's model for Cu‐BTC, ZIF‐8, ZSM‐5, and MIL‐53(Al) or the Virial model for MOF‐177.

**Figure 3:**
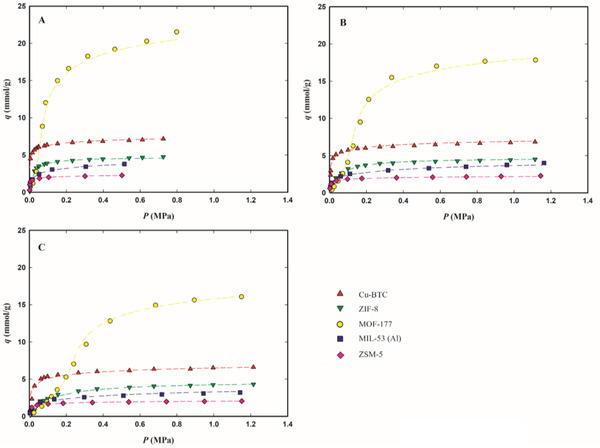
Adsorption equilibrium isotherms of R‐125 on Cu‐BTC (red up triangles), ZIF‐8 (green down triangles), MOF‐177 (yellow circles), MIL‐53 (Al) (blue squares), and zeolite ZSM‐5 (pink diamond), at 283.15 K (panel A), 303.15 K (panel B), and 323.15 K (panel C). The dashed lines represent the data fittings with the Tóth's model for Cu‐BTC, ZIF‐8, ZSM‐5, and MIL‐53(Al) or the Virial model for MOF‐177.

**Figure 4:**
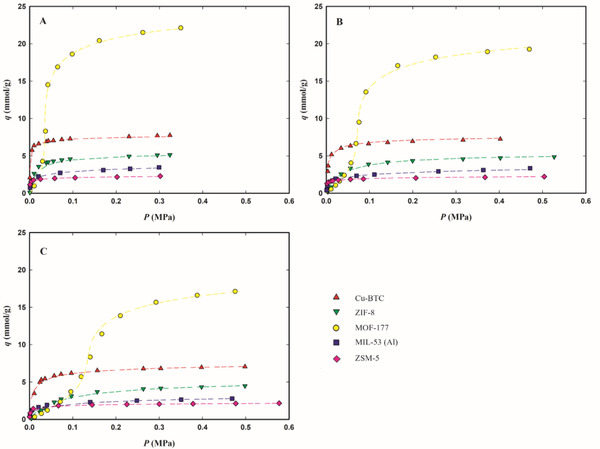
Adsorption equilibrium isotherms of R‐134a on Cu‐BTC (red up triangles), ZIF‐8 (green down triangles), MOF‐177 (yellow circles), MIL‐53 (Al) (blue squares), and zeolite ZSM‐5 (pink diamond), at 283.15 K (panel A), 303.15 K (panel B), and 323.15 K (panel C). The dashed lines represent the data fittings with the Tóth's model for Cu‐BTC, ZIF‐8, ZSM‐5, and MIL‐53(Al) or the Virial model for MOF‐177.

The experimental adsorption equilibrium data at 283.15, 303.15, and 323.15 K, expressed as millimoles of adsorbed gas per gram of adsorbent (mmol g^−1^), are plotted in **Figures** **2**, **3**, **4** as a function of the equilibrium gas pressure (all experimental data are listed in Tables S1–S5, Supporting Information). The maximum adsorption pressure for each studied F‐gas was at least 10% lower than its vapor pressure at each measured temperature. Figure S4 in the Supporting Information presents the isotherms at 303.15 K for the three F‐gases as a function of equilibrium pressure up to 0.15 MPa, to ease the discussion of gas adsorption at low pressures.

Almost no data on the adsorption of F‐gases in the materials studied in this work are available in the literature. Figure S5 in the Supporting Information compares the adsorption of R‐32 and R‐125 in Cu‐BTC at the three studied temperatures (283.15, 303.15, and 323.15 K) and the data available in the literature obtained at 293.15 K; it is shown that there is good agreement and trends between studies.^[^ ^29^ ^]^

All isotherms, except those of MOF‐177, present a classical Type I behavior,^[^ ^45^ ^]^ corresponding to progressive pore filling of gas molecules in microporous adsorbents (see Figure 1). Type V isotherms according to the isotherm classification of International Union of Pure and Applied Chemistry,^[^ ^46^, ^47^, ^48^, ^49^ ^]^ observed for the MOF‐177 (see Figure 1), are typical of mesoporous adsorbents in which the adsorption process is governed by both adsorbent–adsorbate interactions and interactions between the adsorbate molecules in the condensed state.^[^ ^45^ ^]^ The adsorption data were fitted by isotherm models, with good agreement between calculated and experimental data. Considering the behavior of the isotherms, two different adsorption equilibrium models were employed. Experimental adsorption data were fitted using the Tóth's model for the zeolite ZSM‐5 and the MOFs Cu‐BTC, MIL‐53(Al), and ZIF‐8. MOF‐177 has a type V isotherm,^[^ ^47^, ^48^, ^49^ ^]^ and therefore cannot be fitted by a Tóth model (comparison of the Tóth and Virial fittings for R‐32 adsorption in MOF‐177 are shown in Figure S6, Supporting Information). In this case the experimental adsorption data for MOF‐177 were fitted with the Virial isotherm model.^[^ ^48^, ^50^ ^]^ This model is very flexible to adjust isotherms with different degrees of inclination and inflexion points and allows predicting multicomponent adsorption using the virial coefficients of the mixture.^[^ ^46^, ^51^, ^52^ ^]^

The Tóth model^[^ ^53^ ^]^ is described by

(1) $$\begin{array}{c} q=\frac{q_{\infty }bP}{{\left[1+{\left(bP\right)}^{t}\right]}^{\frac{1}{t}}} \end{array}$$

where *q*_∞_ = log*_P_*_→∞_ *q; b = K*_H_ */q*_∞_ is an affinity constant (MPa^−1^); *K*_H_ (mmol g^−1^ MPa^1^) is the Henry constant; and *t* is a dimensionless heterogeneity parameter. The parameters *b* and *t* depend on temperature as follows

(2) $$\begin{array}{c} b=b_{0}\exp\left(\frac{Q}{RT}\right) \end{array}$$

(3) $$\begin{array}{c} t=t_{0}+B\left(1-\frac{T_{0}}{T}\right) \end{array}$$

where *b*_0_ is the affinity constant at infinite temperature; *Q* is the isosteric heat of adsorption; *t*_0_ is the heterogeneity parameter at a reference temperature *T*_0_; and *B* is the slope of the plot of *t* versus −1/*T*.

The Virial isotherms,^[^ ^48^, ^50^ ^]^ truncated after the third virial coefficient, can be expressed as

(4) $$\begin{array}{c} P=\frac{q}{K_{H}}\exp\left(2Aq+\frac{3}{2}Bq^{2}+\frac{4}{3}Cq^{3}\right) \end{array}$$

where *q* is the amount adsorbed; *p* is the partial pressure of the adsorbate; *K*_H_ is Henry's constant; and A, B, and C are the viral coefficients. The Henry's constant is temperature‐dependent and is determined by the van't Hoff equation as follows

(5) $$\begin{array}{c} K_{H}=K_{\infty }\exp\left(\frac{\Delta H}{RT}\right) \end{array}$$

where *K*_∞_ is the constant at infinite temperature, *R* is the ideal gas constant, Δ*H* is the isosteric heat of adsorption, and *T* is the temperature. Viral coefficients *A*, *B*, and *C* have temperature dependence and are truncated after the second term.

(6) $$\begin{array}{c} A=\sum_{m}^{\infty }\frac{A_{m}}{T^{m}} \end{array}$$

(7) $$\begin{array}{c} B=\sum_{m}^{\infty }\frac{B_{m}}{T^{m}} \end{array}$$

(8) $$\begin{array}{c} C=\sum_{m}^{\infty }\frac{C_{m}}{T^{m}} \end{array}$$

The Tóth and Virial parameters that best fit the experimental adsorption data for each adsorbent/F‐gas pair are listed in Tables S6 and S7 in the Supporting Information and the corresponding fittings are plotted in Figures 2, 3, 4 as dashed lines. The chosen models correlate well with the adsorption equilibrium data at low and high pressures on the studied materials, describing well the thermodynamics of the studied F‐gas/adsorbent systems. Therefore, these models are suitable for use in the modeling and simulation of adsorption‐based separation processes.

The micro‐mesoporous MOF‐177 has the highest adsorption capacity of the three studied HFCs, adsorbing more than double the amount of each gas, when compared with the other four microporous materials (see Figures 2, 3, 4). At 303.15 K and the maximum tested pressure for each F‐gas, this material adsorbs around 30 mmol g^−1^ of R‐32 (at 1.30 MPa), 19 mmol g^−1^ of R‐134a (at 0.47 MPa), and 18 mmol g^−1^ of R‐125 (at 1.11 MPa) (see Table S3, Supporting Information). On the other hand, at low pressures, MOF‐177 adsorbs similar or lower amounts of all F‐gases, compared to the other materials (see Figure S7, Supporting Information). This peculiar behavior of MOF‐177 may be explained by a primary interaction of the gas molecules with the adsorbent pore walls at lower pressures, followed by interactions between gas molecules that allow the accommodation of more gas molecules in the mesopores at higher pressures.

**Figure 5:**
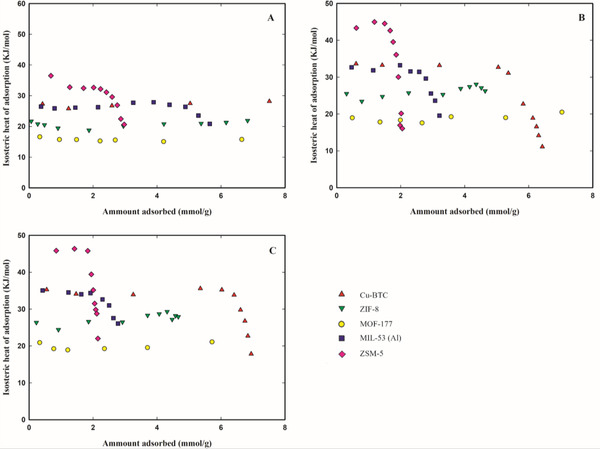
Differential heats of adsorption of R‐32 (panel A), R‐125 (panel B), and R‐134a (panel C) at 323.15 K on Cu‐BTC (red up triangles), ZIF‐8 (green down triangles), MOF‐177 (yellow circles), MIL‐53 (Al) (blue squares), and Zeolite ZSM‐5 (pink diamond).

While for all materials the highest adsorption capacities were achieved for R‐32, at low pressures most of the studied materials adsorb fewer R‐32, when compared to the other two studied gases (see Figure S7, Supporting Information). This discrepancy in the R‐32 adsorption behavior at low and at high pressures is more remarkable for ZIF‐8 and MOF‐177, which are the materials that adsorb the lowest amount of R‐32 at low pressures (below 0.15 MPa in Figure S4, Supporting Information) and the ones with the widest pores and with the highest superficial areas of all studied materials. At low pressures, adsorption is typically governed by enthalpic processes where the strength of the solid–fluid interaction decreases with increasing micropore size. At low amounts of adsorbed gas, ZIF‐8 and MOF‐177 have the lowest values of isosteric heat (see **Figure** **5**), indicating a low degree of interaction between the gas molecules and these two adsorbents. At high pressures and conditions close to pore filling, the entropy of the condensed adsorbed phase controls gas adsorption. Therefore, the saturation capacity, *q*_∞_, is inversely proportional to the molar volume, *v*_m_, of each HFC (*v*_m_ *_:_* R‐32 < R‐134a < R‐125) and directly proportional to pore volume and superficial area available for gas adsorption. MIL‐53(Al) and ZSM‐5, the materials with the lowest superficial areas and with the narrower micropores (<10 Å), have the lowest adsorption capacity for the three studied gases. Interestingly, Cu‐BTC has higher gas adsorption capacity than ZIF‐8 and MIL‐53(Al) (see Figure 2), despite having the smallest *v*_p_ of all MOFs and the second smallest superficial area of all MOFs (see Table 1 and Figure 1). This indicates that for this material, Cu‐BTC, other factors control the gas adsorption capacity.

The differential heats of adsorption (determined for each HFC/adsorbent at 323.15 K) for R‐134a and R‐125 (see Figure 5) are higher than for R‐32, in accordance with the higher heats of vaporization of R‐134a and R‐125. These results may also indicate a lower degree of interaction between R‐32 molecules and the adsorbents, probably because of the existence of fewer hydrogen and fluorine atoms available for interactions. This behavior may explain why R‐32 is preferentially adsorbed at higher pressures. On the other hand, for low sorbate adsorbed amount, the heat of adsorption for the zeolite ZSM‐5 is higher than the other materials, exceeding 30 kJ mol^−1^ for R‐32 and 40 kJ mol^−1^ for R‐125. In this material electrostatic interactions are likely to dominate over weaker dispersive interactions, such as van der Waals forces.^[^ ^14^ ^]^ Concluding, at lower pressures, where the enthalpic factors govern the adsorption, all materials adsorb preferentially R‐125 and R‐134a, but at higher pressures, where the entropic factors lead the process, the materials showed higher adsorption capacity of the gas with the lowest molar volume: R‐32. Furthermore, MOF‐177 is the material with the highest adsorption capacity at higher pressures for the three studied F‐gases, resulting from its mesoporous nature, which allows the packing of more gas molecules in its pores at high pressures.

### 2.3 Prediction of the Adsorption of R‐410A and R‐407F Using the Ideal Adsorption Solution Theory (IAST)

**Figure 6:**
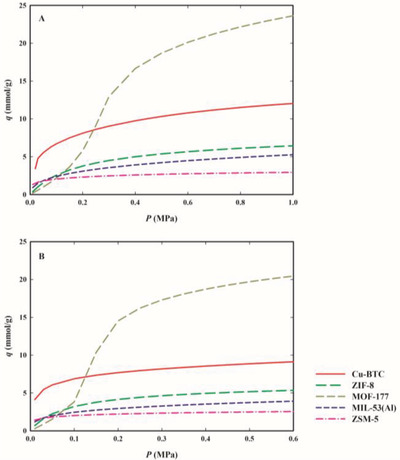
IAST prediction of the adsorbed amounts of R‐410A (panel A) and R‐407F (panel B) as a function of pressure at the equilibrium gas‐phase mole fractions corresponding to the bulk composition (R‐410A: *y*_R‐32_ = 0.7 and *y*_R‐125_ = 0.3 and R‐407F: *y*_R‐32_ = 0.47, *y*_R‐125_ = 0.21, and *y*_R‐134a_ = 0.32) at 303.15 K.

The commercial refrigerants R‐410A (R‐32/R‐125, 50/50 wt%; *y* ^(0)^_R‐32_ = 0.7, *y* ^(0)^_R‐125_ = 0.3) and R‐407F ((R‐32/R‐125/R‐134a, 30/30/40 wt%; *y* ^(0)^_R‐32_ = 0.47, *y* ^(0)^_R‐125_ = 0.21, and *y* ^(0)^_R‐134a_ = 0.32) are near azeotropic blends of HFCs. Therefore, these refrigerants are not easily separated into their individual components by conventional methods, such as distillation. In this work we explored the adsorption of R‐32, R‐125, and R‐134a in five porous materials, aiming at evaluating their capability to split R‐410A and R‐407F into their components. The multicomponent adsorption equilibrium data of R‐410A, R‐407F, and their components were predicted using the IAST^[^ ^54^, ^55^ ^]^ from the Tóth and Virial fittings to the single‐component adsorption equilibrium data of R‐32, R‐125, and R‐134a at 303.15 K. The adsorption equilibria of the gas blends were predicted for pressures up to 1.0 MPa for R‐410A and up to 0.6 MPa for R‐407F, to not exceed the saturation pressure of the individual pure F‐gas at 303.15 K. The results are plotted in **Figure** **6** and values are presented in Tables S8 and S9 in the Supporting Information. The adsorption of both gas blends in all materials increases with increasing pressure and at the highest tested pressures, the highest adsorption capacities were obtained for MOF‐177, which is the studied material with the highest value for *S*_BET_ and *v*_p_ as well as the one with the widest pores. On the other hand, at the lowest tested pressures (*P* < 0.1 MPa), MOF‐177 is the material with the lowest adsorption capacity and Cu‐BTC is the one with the highest capacity. The lowest adsorption capacity for both gas blends, at the highest tested pressures, was obtained for zeolite ZSM‐5, which is the studied material with the lowest values for *S*_BET_ and *v*_p_ as well as the one with the narrowest pores.

### 2.4 Selectivity of the ACs for F‐Gases in Binary and Ternary Mixtures

The selectivity of the materials for the separation of the pure components (R‐32, R‐125, and R‐134a) of the two commercial refrigerants, R‐410A and R‐407F, was calculated using the predictions determined by IAST. The gas selectivity (*S_ij_*) at 303.15 K of each material for the separation of a gas *i* from the mixture with a gas *j* was determined as follows

(9) $$\begin{array}{c} S_{\text{ij}}=\frac{x_{i}/x_{j}}{y_{i}/y_{j}} \end{array}$$

where *x_i_* and *x_j_* are the adsorbed molar fractions of gas *i* and of gas *j*, respectively, and *y_i_* and *y_j_* are the mole fractions of gas *i* and of gas *j* in the gas phase at equilibrium, respectively.

**Figure 7:**
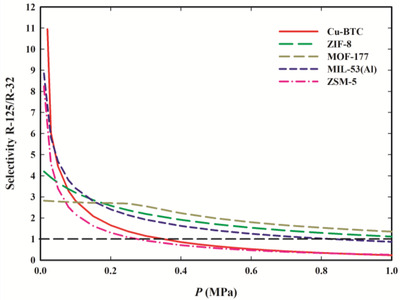
Selectivity of Cu‐BTC (red line), ZIF‐8 (green line), MOF‐177 (yellow line), MIL‐53 (Al) (blue line), and zeolite ZSM‐5 (pink line) to R‐32 over R‐125 as a function of pressure at 303.15 K, for the equilibrium gas phase composition of bulk the R‐410A mixture (*y*_R‐32_ = 0.7 and *y*_R‐125_ = 0.3).

In the case of R‐410A, the selectivity of each material toward R‐125 over R‐32 was determined. The selectivity was determined as a function of pressure for the equilibrium gas‐phase composition corresponding to that of bulk R‐410A. The results are plotted in **Figure** **7** and the values are presented in Table S10 in the Supporting Information. If *S* > 1, R‐125 is preferentially adsorbed but if *S* < 1, R‐32 is preferentially adsorbed.

At pressures below 0.3 MPa, all materials are selective toward R‐125, with Cu‐BTC, MIL‐53(Al), and ZSM‐5 achieving selectivities of around 11, 9, and 8, respectively, at the lowest studied pressures. Interestingly, at higher pressures (above 0.4 MPa), the selectivity of Cu‐BTC and ZSM‐5 is shifted toward R‐32 (reaching selectivities for R‐32 of around 2 at 1.0 MPa). On the other side, MOF‐177, ZIF‐8, and MIL‐53(Al) have no significant selectivity toward any of the two gases at high pressures. In this case, the dimensions of the pores seem to play an important role in the selectivity. The materials with the narrowest pores have higher selectivity toward R‐125 at low pressures, probably as a result of higher interaction of the R‐125 molecules (which have a higher number of fluorine atoms to establish interactions) with the pore walls. At high pressures, the selectivity of Cu‐BTC and ZSM‐5 is shifted toward R‐32, possibility due to the smaller size of this molecule, which facilitates its packing at the smaller pores.

**Figure 8:**
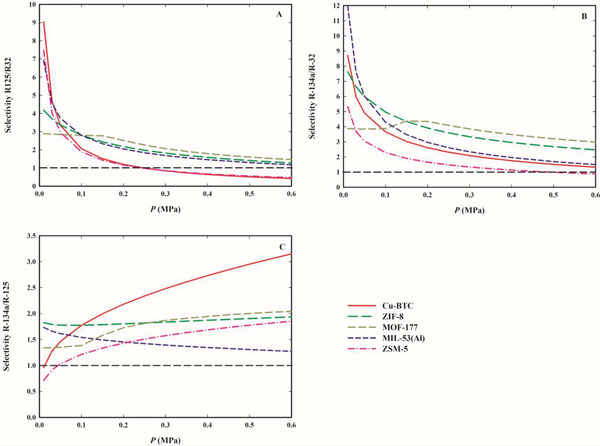
Selectivities of Cu‐BTC, ZIF‐8, MOF‐177, MIL‐53(Al), and ZSM‐5 to: R‐125 over R‐32 (panel A); R‐134a over R‐32 (panel B); and R‐134a over R‐125 (panel C), for the commercial R‐407F gas mixture (*y*_R‐32_ = 0.47, *y*_R‐125_ = 0.21, and *y*_R‐134a_ = 0.32), as a function of pressure at 303.15 K.

In the case of the R‐407F blend (ternary mixture of R‐32, R‐125, and R‐134a), the selectivities for the separations of R‐125/R‐32, R‐134a/R‐125, and R‐134a/R‐32 were determined as a function of pressure at 303.15 K for the equilibrium gas‐phase composition of the bulk R‐407F. The results are shown in **Figure** **8** and the values are presented in Table S11 in the Supporting Information.

In general, all materials are selective for the separation of R‐134a from mixtures with the other two gases (*S*_R134a/R32_ > 1 and *S*_R134a/R125_ > 1). While *S*_R‐134a/R‐32_ decreases with pressure for all materials, *S*_134a/R‐125_ increases with the pressure for Cu‐BTC, MOF‐177, and ZMS‐5; decreases with pressure for MIL‐53(Al); and is kept almost constant for ZIF‐8. Concluding, the textural properties of materials play an important role in defining the selectivity toward the different studied F‐gases, with all materials being selective toward R‐125 and R‐134a over R‐32 at low pressures. For Cu‐BTC and ZSM‐5, the results show that R‐125/R‐32 selectivity (in the binary and ternary blends) can be shifter either toward R‐125 or to R‐32 by changing the pressure.

## 3 Conclusion

The emergence of new materials, such as MOFs, brings new and promising perspectives for the separation of refrigerant blends. Since they are excellent tailor‐made materials, thousands of different MOFs can be designed and prepared.

In this work, four MOFs with different metal sites, chemical compositions, and textural properties, and one zeolite were selected and studied for the selective adsorption of three pure F‐gases (R‐32, R‐125, and R‐134a) at three different temperatures (283.15, 303.15, and 323.15 K) at a range of pressures up to 1.4 MPa. For the four tested microporous materials (three MOFs and one zeolite), the type I isotherms were successfully correlated with the Tóth equation, while for the micro‐mesoporous MOF‐177 the obtained type V isotherms were successfully correlated with the Virial model. At lower pressures, R‐125 and R‐134a are preferentially adsorbed in all materials, probably due to enthalpic factors resulting from a higher degree of interactions between the gas molecules and the adsorbent. However, at higher pressures, entropic factors may be involved in the gas adsorption process and therefore the selectivity is shifted toward the adsorption of the gas with the lowest molar volume (R‐32), or toward a value of 1 (no selectivity). Furthermore, MOF‐177 is the material with the highest adsorption capacity at higher pressures for the three studied F‐gases, resulting from its mesoporous nature, which allows the packing of more gas molecules in its pores at high pressures.

The results presented in this work show that the utilization of MOFs, as tailored made materials, is promising for the development of new approaches for the selective capture of F‐gases and for the separation of blends of these gases, which are used in commercial refrigeration. Moreover, this work contributes to the fundamental understanding of the behavior of these novel materials and the different interactions and mechanisms involved in gas adsorption. Finally, this work opens possibilities for the development of improved materials.

## 4 Experimental Section

### Materials

Difluoromethane, R‐32 (wt% purity ≥ 99.9) and pentafluoroethane, R‐125 (wt% purity ≥ 99.8) were supplied by Polo Zero (Portugal). 1,1,1,2‐tetrafluoroethane, R‐134a (wt% purity ≥ 99.8), was supplied by Air liquid (Portugal). The designation and chemical structure of these F‐gases are shown in **Table** **2**. Nitrogen (N_2_) (wt% purity ≥ 99.9999) was supplied by Linde Gas (France) and Helium (wt% purity ≥ 99.999) by Praxair (Portugal). For this work one zeolite and four MOFs were purchased: zeolite ZSM‐5 (Alfa Aesar, Germany); ZIF‐8 (Basolite Z1200, Sigma‐Aldrich, Portugal); MIL‐53(Al) (Basolite A100, Sigma‐Aldrich, France), Cu‐BTC (HKUST‐1 or MOF‐199, MOF Technologies, UK); and MOF‐177 (Basolite Z377, Sigma‐Aldrich, France).

**Table 2:** Chemical structures and properties (vapor pressure, *P*_s_; critical volume, *V*_c_; and critical temperature, *T*_c_) of the HFCs studied in this work

Gas	Chemical structure	*P*_s_ ^a)^ [MPa]	*V*_c_ [cm^3^ mol^−1^]	*T*_c_ [K]
R‐32 (difluoromethane)	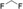	1.93	120.8	351.26
R‐125 (pentafluoroethane)	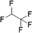	1.57	211.3	339.17
R‐134a (1,1,1,2‐tetrafluoroethane)	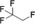	0.77	198.8	374.21

^a)^ At 303.15 K.

### Experimental Procedure

The textural properties of the adsorbents were determined using a gas porosimeter from Quantachrome (Autosorb iQ). Argon (Ar) adsorption at 87 K and nitrogen (N_2_) adsorption at 77 K (Figure S1, Supporting Information) were determined for the zeolite and MOFs, respectively. Furthermore, the pore volume, the specific BET surface area (*S*_BET_), and the PSD (nonlocalized density functional (NLDFT) method)^[^ ^56^ ^]^ were calculated from these adsorption isotherms.

The single‐component adsorption equilibrium isotherms for R‐32, R‐125, and R‐134a were measured using a standard gravimetric method at 283.15, 303.15, and 323.15 K, and at pressures up to a *p*/*p* ^0^ ratio of 0.9, where *p* ^0^ is the vapor pressure of the adsorptive at the imposed temperature. These adsorption measurements^[^ ^57^, ^58^, ^59^ ^]^ were performed in a high‐accuracy IsoSORP high‐pressure magnetic‐suspension balance (Rubotherm GmbH, Germany) with a maximum loading of 25 g, resolution of 10 µg, uncertainty less than 0.002%, and reproducibility  smaller than  3 × 10^−5^ g. The experimental setup is schematized in Figure S2 in the Supporting Information. This method consists of the stepwise addition of pure gas to a temperature‐controlled cell containing the adsorbent (≈0.4 g), while the mass and pressure variations are monitored until equilibrium is reached (i.e., when pressure and mass changes in the system are no longer detected). The pressure was measured with transducers working with accuracy over the full range of operating pressures: a Baratron model 627D (MKS Instruments GmbH, Germany) for 0–0.1 MPa, with the accuracy of 0.12% of the measured value, and two Omegadyne Inc. (Sunbury, OH, USA) models PX01C1‐150A5T and PX01C1‐500A5T, respectively for 0–1 and 0–3.5 MPa (both with the accuracy of 0.05% of the full scale). Before the measurements, the adsorbent samples were degassed for at least 12 h under vacuum at a minimum of 373.15 K.

The adsorption equilibria data are reported in terms of the amount of adsorbed gas per mass of adsorbent (*q*, mmol g^−1^):

(10) $$\begin{array}{c} q=\frac{q_{\text{ex}}}{1-v_{a}\rho_{g}} \end{array}$$

where *v*_a_ (cm^3^ mol^−1^) is the molar volume of the adsorbed phase, which was approximated to the molar volume, *v*_m_, of the saturated liquid adsorptive at the adsorption temperature (for subcritical adsorption);^[^ ^60^ ^]^ ρ_g_(T,P) is the gas density at the equilibrium pressure and temperature of the experiment and whose value was extracted from the National Institute of Standards and Technology Chemistry Webbook;^[^ ^61^ ^]^ and *q*_ex_ is the excess adsorption defined as

(11) $$\begin{array}{c} q_{\text{ex}}=\frac{w-m_{s}+V_{h}\rho_{g}}{m_{s}}+v_{s}\rho_{g} \end{array}$$

where *w* is the apparent mass weighed by the balance, *m*_s_ is the degassed mass of the adsorbent sample in the measurement cell, *V*_h_ is the cumulative volume of all physical parts in the measuring cell contributing to buoyancy effects, and versus is the specific volume of the adsorbent impenetrable to the adsorbate (versus = 1/ρ_s_), where ρ_s_ is the density of the solid matrix of the adsorbent). The solid density ρ_s_ was determined experimentally by helium (He) picnometry.^[^ ^9^, ^58^ ^]^ The uncertainty of the calculated adsorbed mass is around 1% over the full range of temperature and pressure.^[^ ^62^ ^]^

The differential heat of adsorption, *Q*_diff_, as a function of the equilibrium amount of gas adsorbed at 223.15 K for each adsorbent was determined using a homemade apparatus coupling manometric and calorimetric techniques, as described in refs. [9, 63] and whose scheme is shown in Figure S3 in the Supporting Information. The overall uncertainty^[^ ^63^ ^]^ in the measurements of *Q*_diff_ is less than 5%.

## Conflict of Interest

The authors declare no conflict of interest.

## Supporting information

Supporting Information

## Data Availability

The data that support the findings of this study are available from the corresponding author upon reasonable request.
