# Supplementary material for: Exploring the Potential of Metal–Organic Frameworks for the Separation of Blends of Fluorinated Gases with High Global Warming Potential
Source: Glob Chall. 2022 Oct 21;7(1):2200107. doi: 10.1002/gch2.202200107 (PMC9818065; doi:10.1002/gch2.202200107)
Supplement: Supplementary file 1 — Supporting Information [file GCH2-7-2200107-s001.pdf]

Global Challenges
Open Access

Supporting Information

for *Global Challenges,* DOI: 10.1002/gch2.202200107

Exploring the Potential of Metal–Organic Frameworks for the Separation of Blends of Fluorinated Gases with High Global Warming Potential

*Julio E. Sosa, Carine Malheiro, Paulo J. Castro, Rui P. P. L. Ribeiro, Manuel M. Piñeiro, Frédéric Plantier, José P. B. Mota, João M. M. Araújo, and Ana B. Pereiro**

SUPPORTING INFORMATION

Exploring the Potential of Metal-Organic Frameworks for the Separation of Blends of Fluorinated Gases with High Global Warming Potential

Julio E. Sosa[a], Carine Malheiro[b], Paulo J. Castro[a], Rui P. P. L. Ribeiro[a], Manuel M. Piñeiro[c], Frédéric Plantier[b], José P. B. Mota[a], João M. M. Araújo[a], and Ana B. Pereiro*[a]

[a] J. E. Sosa, P.J. Castro, R.P.P.L. Ribeiro, J.P.B. Mota, J.M.M.Araújo, A.B. Pereiro
Department of Chemistry
LAQV, REQUIMTE
NOVA School of Science and Technology, NOVA University Lisbon, 2829-516 Caparica, Portugal
E-mail: anab@fct.unl.pt

[b] C. Malheiro, F.Plantier
Université de Pau et des Pays de l'Adour
E2S UPPA, CNRS, TOTAL, LFCR, Anglet, France

[c] M.M. Piñeiro
Departamento de Física Aplicada
Facultade de Ciencias, Universidade de Vigo
E36310 Vigo, Spain

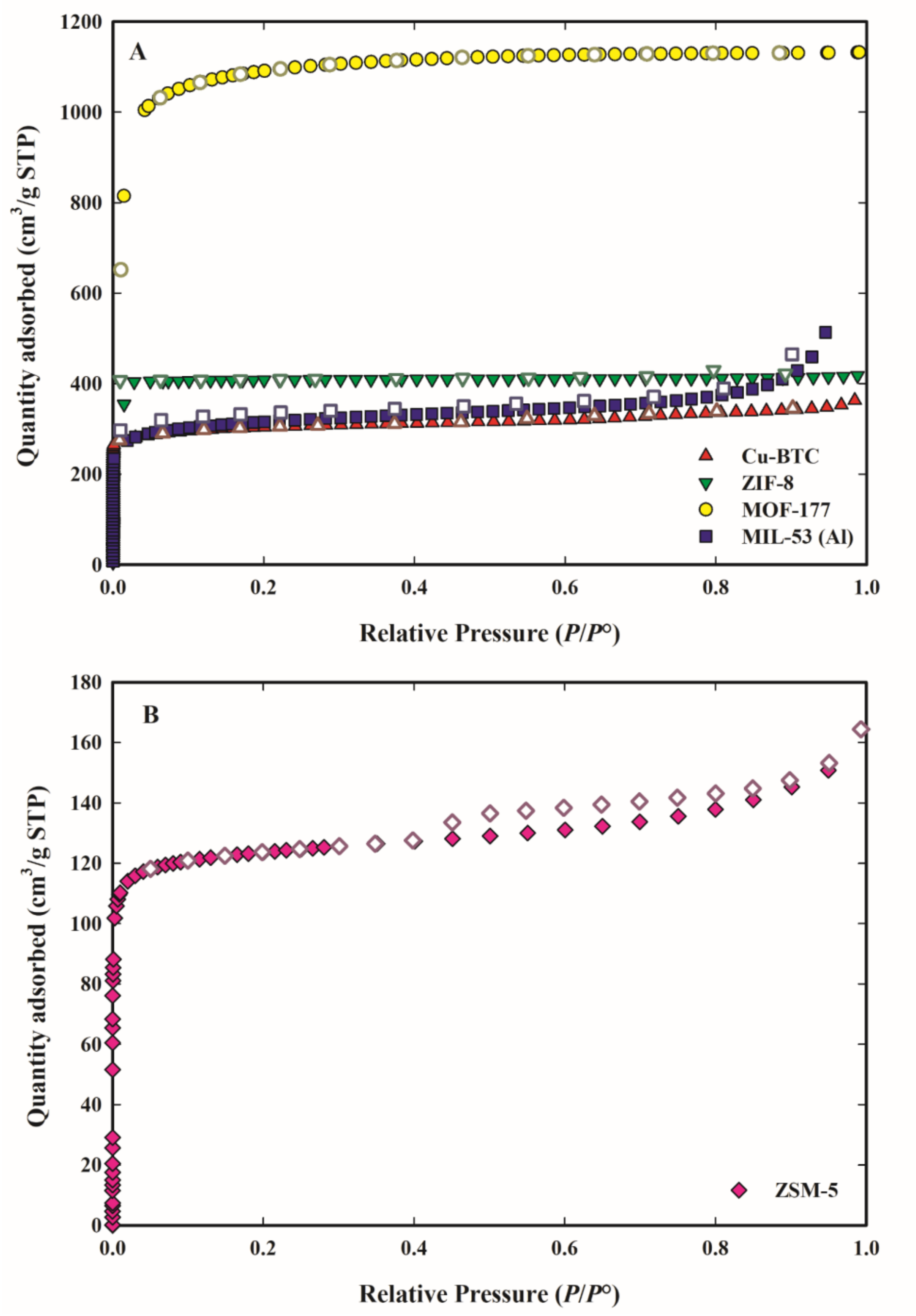

Figure S1. Adsorption isotherms of nitrogen at 77 K for Cu-BTC, ZIF-8, MOF 177 and MIL 53 (Al) (panel A), and of argon at 87 K for zeolite ZSM5 (panel B).

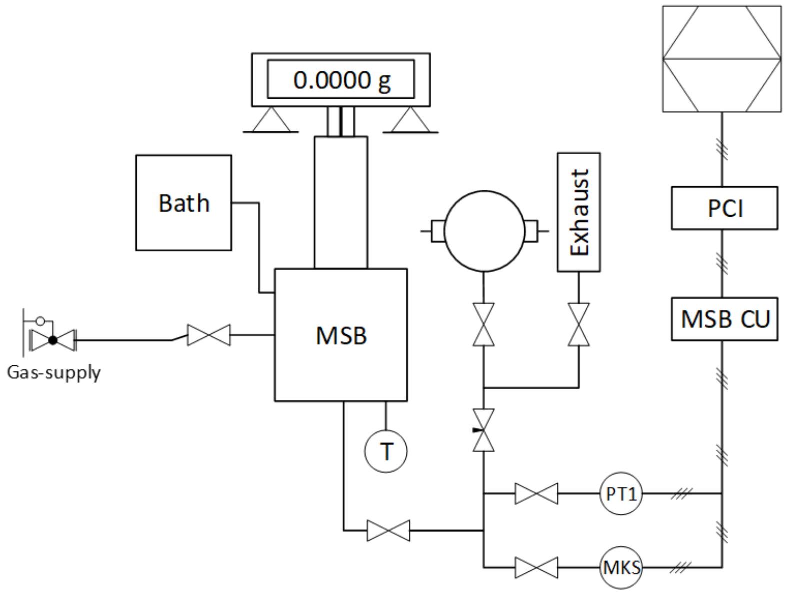

Figure S2. Experimental set-up of the magnetic suspension microbalance where PCI is the PC interface for data acquisition, T is the Pt100 temperature sensor, PT represents the Omegadyne pressure transducer, and MKS is the MKS Baratron pressure transducer.

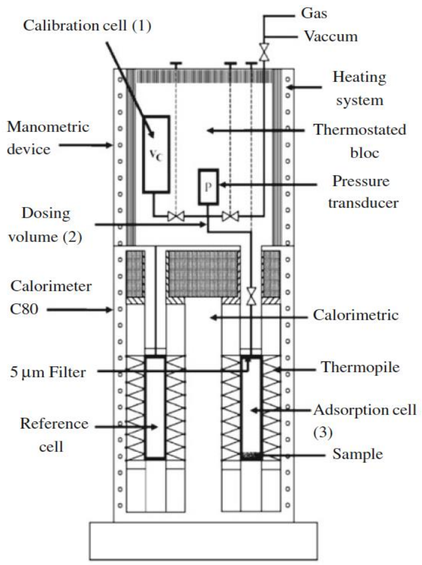

Figure S3. Schematic diagram of the manometric/calorimetric device.

Table S1. Adsorption Isotherms on Cu-BTC. Pressure, *P*, in MPa, and Equilibrium Loadings, *q*, in mmol/g.

R-32					
283.15 K		303.15 K		323.15 K	
P **(MPa)**	***q*** **(mmol/g)**	***P*** **(MPa)**	***q*** **(mmol/g)**	***P*** **(MPa)**	***q*** **(mmol/g)**
0.004	1.47	0.009	0.921	0.011	0.631
0.012	4.73	0.016	1.99	0.018	1.17
0.023	7.11	0.027	3.54	0.033	2.04
0.033	8.53	0.027	4.15	0.051	3.24
0.040	9.44	0.048	5.75	0.055	3.64
0.059	10.5	0.053	6.36	0.087	5.23
0.070	10.9	0.076	7.74	0.159	7.55
0.081	11.2	0.100	8.58	0.182	8.01
0.089	11.3	0.158	9.99	0.252	9.12
0.099	11.5	0.197	10.5	0.288	9.42
0.109	11.7	0.271	11.1	0.451	10.5
0.146	12.0	0.378	11.7	0.639	11.1
0.222	12.6	0.522	12.1	0.836	11.6
0.315	13.0	0.776	12.5	1.031	11.8
0.462	13.3	0.919	12.7	1.118	12.0
0.535	13.5	1.120	12.9	1.345	12.2
0.684	13.7	1.209	13.0	-	-
0.770	13.9	1.313	13.1	-	-
0.851	14.1	-	-	-	-

R-134a					
283.15 K		303.15 K		323.15 K	
P **(MPa)**	***q*** **(mmol/g)**	***P*** **(MPa)**	***q*** **(mmol/g)**	***P*** **(MPa)**	***q*** **(mmol/g)**
0.001	2.08	0.004	2.91	0.004	1.17
0.006	5.76	0.004	3.65	0.011	3.46
0.010	6.37	0.012	5.17	0.024	4.99
0.021	6.62	0.034	6.02	0.027	5.31
0.038	6.90	0.056	6.32	0.036	5.44
0.043	7.00	0.098	6.62	0.057	5.83
0.055	7.06	0.142	6.78	0.073	6.04
0.074	7.18	0.200	6.94	0.097	6.19
0.094	7.28	0.316	7.11	0.157	6.53
0.230	7.58	0.402	7.24	0.263	6.78
0.295	7.69	0.527	7.35	0.304	6.82
0.324	7.75	-	-	0.398	6.96
-	-	-	-	0.498	7.05

R-125					
**283.15 K**		**303.15 K**		**323.15 K**	
***P* (MPa)**	***q* (mmol/g)**	***P* (MPa)**	***q* (mmol/g)**	***P* (MPa)**	***q* (mmol/g)**
0.002	1.20	0.005	2.39	0.006	0.967
0.005	4.50	0.007	2.99	0.014	2.33
0.016	5.31	0.018	4.62	0.028	4.09
0.026	5.70	0.035	5.12	0.063	5.05
0.038	5.89	0.035	5.16	0.080	5.22
0.049	6.11	0.064	5.51	0.099	5.36
0.051	6.03	0.100	5.74	0.155	5.58
0.080	6.22	0.151	5.95	0.266	5.84
0.095	6.37	0.195	6.01	0.367	6.03
0.152	6.53	0.273	6.18	0.543	6.15
0.233	6.68	0.343	6.23	0.675	6.34
0.324	6.78	0.459	6.35	0.873	6.36
0.397	6.84	0.573	6.48	0.996	6.52
0.540	6.95	0.693	6.57	1.215	6.60
0.612	7.04	0.815	6.65	-	-
0.725	7.17	0.981	6.74	-	-
-	-	1.114	6.85	-	-

Table S2. Adsorption Isotherms on ZIF-8. Pressure, *P*, in MPa, and Equilibrium Loadings, *q*, in mmol/g.

R-32					
283.15 K		303.15 K		323.15 K	
***P* (MPa)**	***q* (mmol/g)**	***P* (MPa)**	***q* (mmol/g)**	***P* (MPa)**	***q* (mmol/g)**
0.004	0.0759	0.009	0.0856	0.011	0.0757
0.012	0.226	0.016	0.172	0.018	0.147
0.023	0.451	0.027	0.292	0.033	0.290
0.033	0.904	0.027	0.400	0.051	0.433
0.040	0.970	0.048	0.590	0.055	0.488
0.059	1.70	0.053	0.746	0.087	0.897
0.070	2.22	0.076	1.08	0.159	1.51
0.081	2.82	0.1	1.53	0.182	1.59
0.089	3.19	0.158	2.53	0.252	2.42
0.099	3.68	0.197	3.35	0.288	2.78
0.109	4.34	0.271	4.52	0.451	4.19
0.146	5.36	0.378	5.60	0.639	5.37
0.222	6.72	0.522	6.41	0.836	6.02
0.315	7.50	0.776	7.22	1.031	6.56
0.462	8.14	0.919	7.55	1.118	6.76
0.535	8.39	1.12	7.89	1.345	7.11
0.684	8.75	1.209	8.05	-	-
0.770	8.94	1.313	8.17	-	-
0.851	9.07	-	-	-	-
**R-134a**					
**283.15 K**		**303.15 K**		**323.15 K**	
***P* (MPa)**	***q* (mmol/g)**	***P* (MPa)**	***q* (mmol/g)**	***P* (MPa)**	***q* (mmol/g)**
0.001	0.0886	0.004	0.194	0.004	0.120
0.006	1.06	0.004	0.301	0.011	0.365
0.010	2.65	0.012	0.824	0.024	0.934
0.021	3.58	0.034	2.57	0.027	1.18
0.038	4.08	0.056	3.33	0.036	1.49
0.043	4.19	0.098	3.87	0.057	2.31
0.055	4.29	0.142	4.14	0.073	2.71
0.074	4.45	0.200	4.34	0.097	3.13
0.094	4.57	0.316	4.57	0.157	3.70
0.230	4.95	0.402	4.71	0.263	4.08
0.295	5.10	0.527	4.85	0.304	4.16
0.324	5.14	-	-	0.398	4.34
-	-	-	-	0.498	4.48

R-125					
283.15 K		303.15 K		323.15 K	
P (MPa)	*q* (mmol/g)	*P* (MPa)	*q* (mmol/g)	*P* (MPa)	*q* (mmol/g)
0.002	0.112	0.005	0.307	0.006	0.182
0.005	0.570	0.007	0.379	0.014	0.402
0.016	1.74	0.018	1.09	0.028	0.849
0.026	2.85	0.035	2.00	0.063	1.78
0.038	3.24	0.035	1.97	0.080	2.11
0.049	3.51	0.064	2.65	0.099	2.38
0.051	3.50	0.100	3.23	0.155	2.94
0.080	3.77	0.151	3.55	0.266	3.41
0.095	3.89	0.195	3.70	0.367	3.68
0.152	4.09	0.273	3.94	0.543	3.89
0.233	4.24	0.343	3.99	0.675	4.05
0.324	4.38	0.459	4.12	0.873	4.14
0.397	4.45	0.573	4.22	0.996	4.24
0.540	4.59	0.693	4.28	1.215	4.31
0.612	4.66	0.815	4.34	-	-
0.725	4.75	0.981	4.42	-	-
-	-	1.114	4.51	-	-

Table S3. Adsorption Isotherms on MOF-177. Pressure, *P*, in MPa, and Equilibrium Loadings, *q*, in mmol/g.

R-32					
283.15 K		303.15 K		323.15 K	
***P* (MPa)**	***q* (mmol/g)**	***P* (MPa)**	***q* (mmol/g)**	***P* (MPa)**	***q* (mmol/g)**
0.043	0.856	0.023	0.295	0.111	0.948
0.111	2.53	0.061	0.782	0.168	1.48
0.167	5.02	0.121	1.64	0.237	2.22
0.212	10.8	0.164	2.35	0.277	2.69
0.233	16.2	0.242	4.08	0.381	4.20
0.279	22.2	0.311	6.57	0.500	6.65
0.325	26.2	0.375	10.8	0.614	10.3
0.498	30.8	0.430	16.3	0.697	13.9
0.628	32.2	0.516	22.2	0.801	17.9
0.762	33.2	0.664	25.9	0.970	21.7
0.887	34.1	0.894	28.3	1.162	23.9
0.985	34.6	1.125	29.5	1.312	25.0
-	-	1.299	30.0	-	-
**R-134a**					
**283.15 K**		**303.15 K**		**323.15 K**	
***P* (MPa)**	***q* (mmol/g)**	***P* (MPa)**	***q* (mmol/g)**	***P* (MPa)**	***q* (mmol/g)**
0.011	0.962	0.011	0.534	0.012	0.332
0.021	2.13	0.022	1.06	0.028	0.774
0.030	4.26	0.031	1.60	0.041	1.21
0.037	8.29	0.042	2.39	0.071	2.36
0.042	14.5	0.057	4.05	0.096	3.70
0.065	16.9	0.069	6.64	0.120	5.72
0.098	18.6	0.076	9.49	0.140	8.34
0.161	20.4	0.092	13.5	0.167	11.4
0.262	21.5	0.165	17.1	0.210	13.9
0.349	22.1	0.253	18.2	0.293	15.7
-	-	0.373	18.9	0.388	16.6
-	-	0.470	19.3	0.476	17.1
**R-125**					
**283.15 K**		**303.15 K**		**323.15 K**	
***P* (MPa)**	***q* (mmol/g)**	***P* (MPa)**	***q* (mmol/g)**	***P* (MPa)**	***q* (mmol/g)**
0.019	1.20	0.013	0.437	0.025	0.485
0.038	2.77	0.025	0.824	0.068	1.35
0.070	8.84	0.047	1.58	0.095	1.99
0.088	12.0	0.072	2.61	0.121	2.67
0.152	15.0	0.099	4.11	0.151	3.58
0.212	16.6	0.128	6.29	0.199	5.28

0.316	18.3	0.166	9.51	0.240	7.04
0.463	19.2	0.212	12.6	0.308	9.68
0.635	20.3	0.337	15.5	0.438	12.8
0.798	21.5	0.580	17.0	0.684	14.9
-	-	0.843	17.7	0.895	15.6
-	-	1.116	17.8	1.150	16.1

Table S4. Adsorption Isotherms on MIL-53(Al). Pressure, *P*, in MPa, and Equilibrium Loadings, *q*, in mmol/g.

**R-32**					
**283.15 K**		**303.15 K**		**323.15 K**	
***P* (MPa)**	***q* (mmol/g)**	***P* (MPa)**	***q* (mmol/g)**	***P* (MPa)**	***q* (mmol/g)**
0.008	0.828	0.007	0.437	0.011	0.374
0.017	1.59	0.015	0.858	0.024	0.795
0.042	2.57	0.028	1.43	0.051	1.44
0.087	3.53	0.050	2.11	0.090	2.16
0.192	4.53	0.114	3.12	0.206	3.26
0.313	5.25	0.275	4.24	0.333	3.89
0.468	5.85	0.485	5.04	0.480	4.39
0.654	6.39	0.702	5.65	0.679	4.88
0.877	7.08	0.921	6.16	0.895	5.29
-	-	1.157	6.71	1.120	5.65
**R-134a**					
**283.15 K**		**303.15 K**		**323.15 K**	
***P* (MPa)**	***q* (mmol/g)**	***P* (MPa)**	***q* (mmol/g)**	***P* (MPa)**	***q* (mmol/g)**
0.001	0.745	0.001	0.400	0.001	0.424
0.003	1.32	0.002	0.986	0.006	1.23
0.020	2.23	0.022	1.93	0.021	1.63
0.071	2.72	0.070	2.32	0.040	1.92
0.170	3.09	0.111	2.49	0.140	2.30
0.232	3.27	0.259	2.92	0.248	2.50
0.299	3.45	0.363	3.10	0.351	2.64
-	-	0.471	3.34	0.468	2.78
**R-125**					
**283.15 K**		**303.15 K**		**323.15 K**	
***P* (MPa)**	***q* (mmol/g)**	***P* (MPa)**	***q* (mmol/g)**	***P* (MPa)**	***q* (mmol/g)**
0.003	1.10	0.004	0.757	0.003	0.462
0.014	1.72	0.012	1.36	0.011	1.14
0.052	2.43	0.060	2.21	0.058	1.98
0.123	3.05	0.110	2.54	0.135	2.30
0.305	3.43	0.318	3.02	0.297	2.58
0.515	3.78	0.539	3.31	0.510	2.78
-	-	0.740	3.51	0.718	2.94
-	-	0.961	3.75	0.944	3.07

Table S5. Adsorption Isotherms on ZSM-5. Pressure, *P*, in MPa, and Equilibrium Loadings, *q*, in mmol/g.

		R-32			
283.15 K		303.15 K		323.15 K	
P (MPa)	*q* (mmol/g)	*P* (MPa)	*q* (mmol/g)	*P* (MPa)	*q* (mmol/g)
0.002	1.23	0.006	0.982	0.006	0.682
0.004	1.51	0.031	1.82	0.021	1.27
0.009	1.76	0.100	2.28	0.046	1.70
0.053	2.30	0.162	2.44	0.089	2.02
0.101	2.59	0.251	2.57	0.150	2.23
0.204	2.78	0.425	2.74	0.248	2.42
0.302	2.92	0.706	2.90	0.428	2.60
0.418	2.99	0.995	3.05	0.702	2.76
0.592	3.11	1.326	3.21	0.994	2.87
-	-	-	-	1.330	2.97
		**R-134a**			
**283.15 K**		**303.15 K**		**323.15 K**	
***P* (MPa)**	***q* (mmol/g)**	***P* (MPa)**	***q* (mmol/g)**	***P* (MPa)**	***q* (mmol/g)**
0.001	1.17	0.002	1.29	0.004	0.344
0.004	1.59	0.005	1.47	0.002	0.850
0.009	1.75	0.011	1.62	0.009	1.42
0.025	1.89	0.015	1.66	0.067	1.83
0.058	1.99	0.028	1.75	0.145	1.95
0.105	2.07	0.056	1.85	0.225	2.01
0.202	2.18	0.086	1.91	0.300	2.05
0.302	2.29	0.207	2.03	0.378	2.08
-	-	0.367	2.13	0.477	2.12
-	-	0.504	2.21	0.577	2.15
		**R-125**			
**283.15 K**		**303.15 K**		**323.15 K**	
***P* (MPa)**	***q* (mmol/g)**	***P* (MPa)**	***q* (mmol/g)**	***P* (MPa)**	***q* (mmol/g)**
0.001	0.283	0.003	0.588	0.003	0.612
0.003	1.02	0.009	1.19	0.014	1.18
0.007	1.47	0.037	1.65	0.043	1.50
0.015	1.63	0.098	1.84	0.102	1.67
0.054	1.87	0.175	1.93	0.184	1.76
0.104	2.01	0.363	2.04	0.342	1.86
0.301	2.16	0.559	2.11	0.539	1.93
0.501	2.26	0.742	2.16	0.743	1.98
-	-	0.931	2.21	0.949	2.02
-	-	1.144	2.28	1.152	2.05

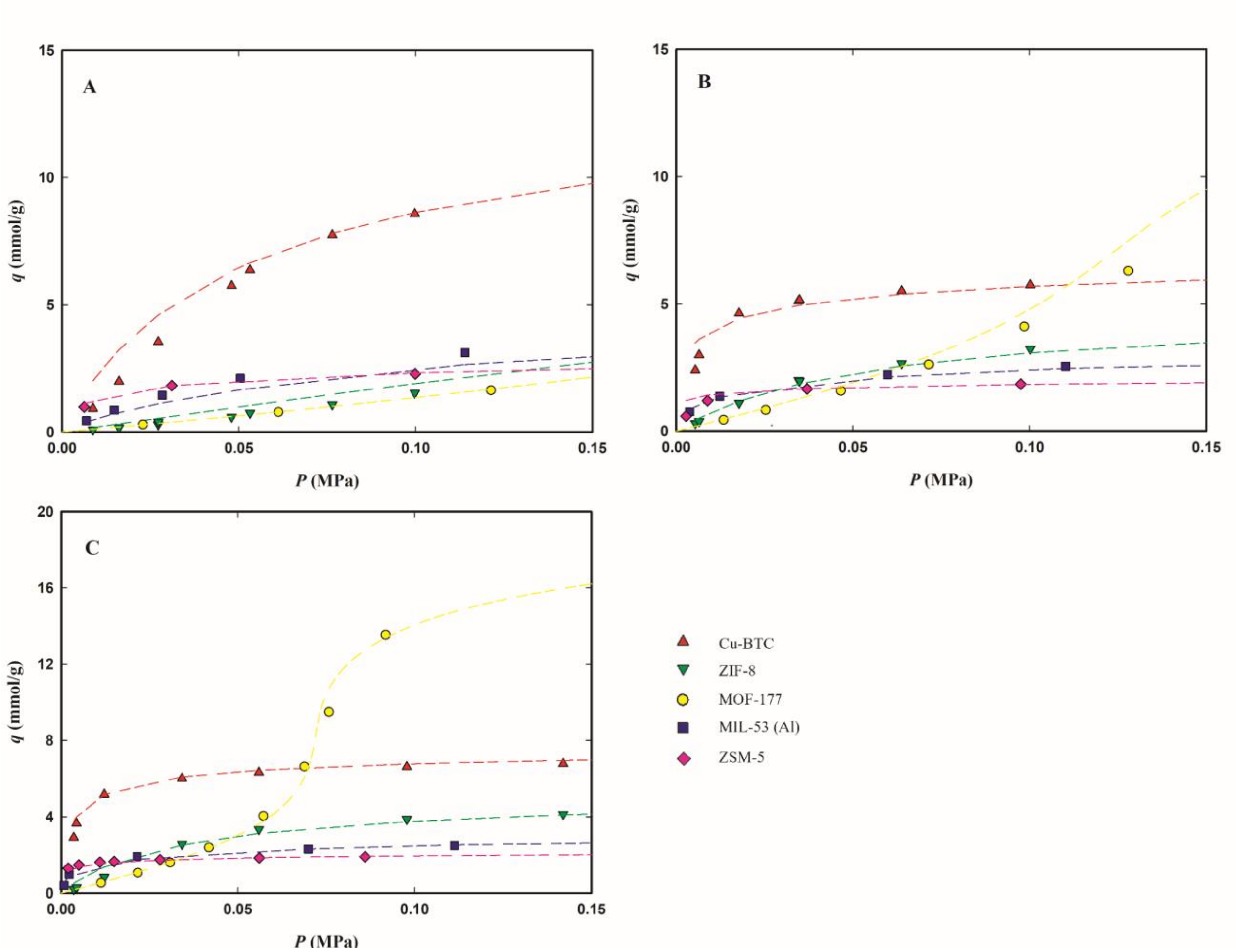

Figure S4. Adsorption equilibrium isotherms at 303.15 K of R-32 (panel A), R-125 (panel B), and R-134a (panel C) on Cu-BTC (red up triangles), ZIF-8 (green down triangles), MOF-177 (yellow circles), MIL-53 (Al) (blue squares), and zeolite ZSM-5 (pink diamond). The dashed lines represent the fittings with the Tóth's or Virial models.

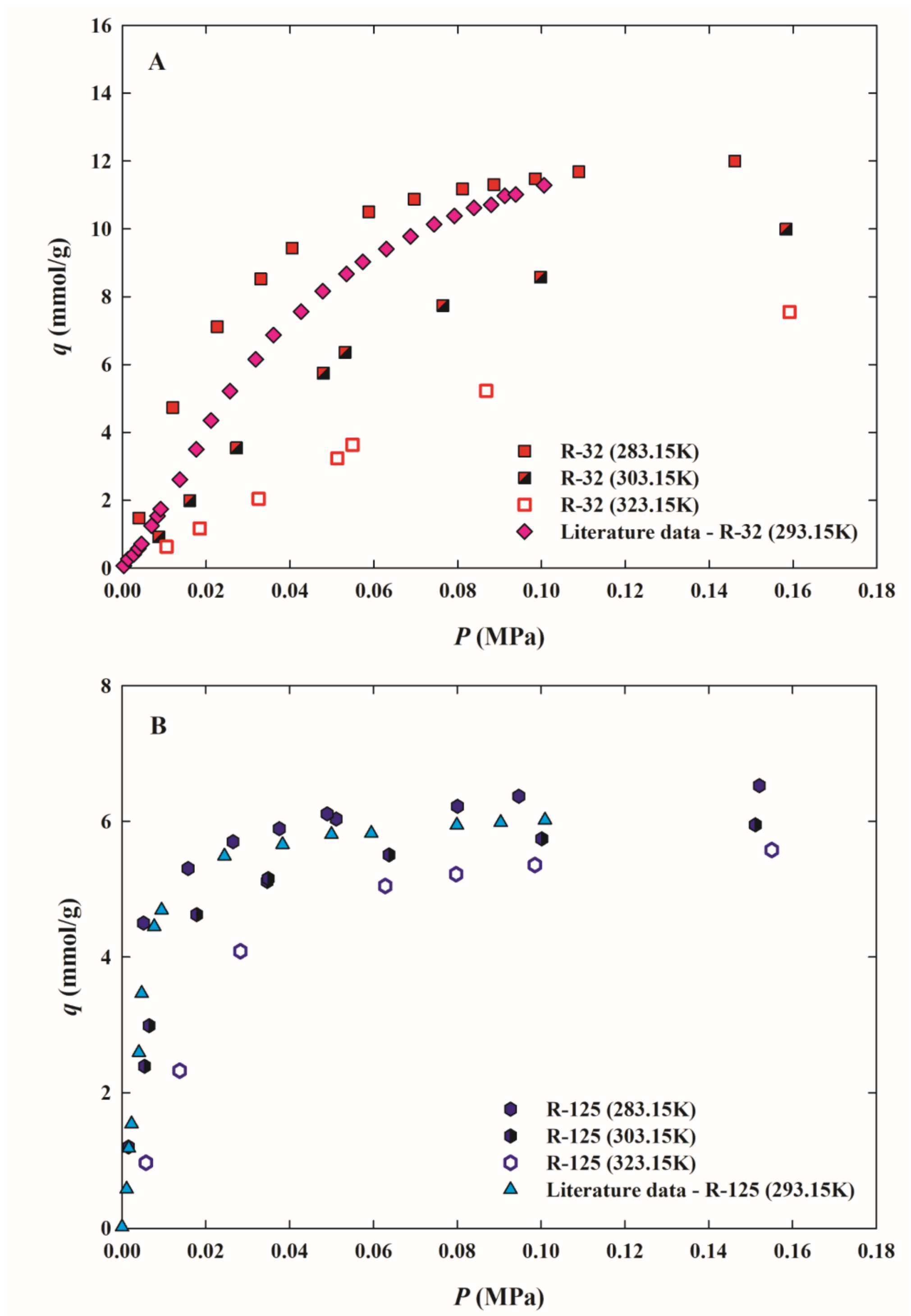

Figure S5. Comparison between the adsorption of R-32 (panel A) and R-125 (panel B) in Cu-BTC at 283.15, 303.15, and 323.15 K, studied in this work, and literature data available at 293.15 K.[S1]

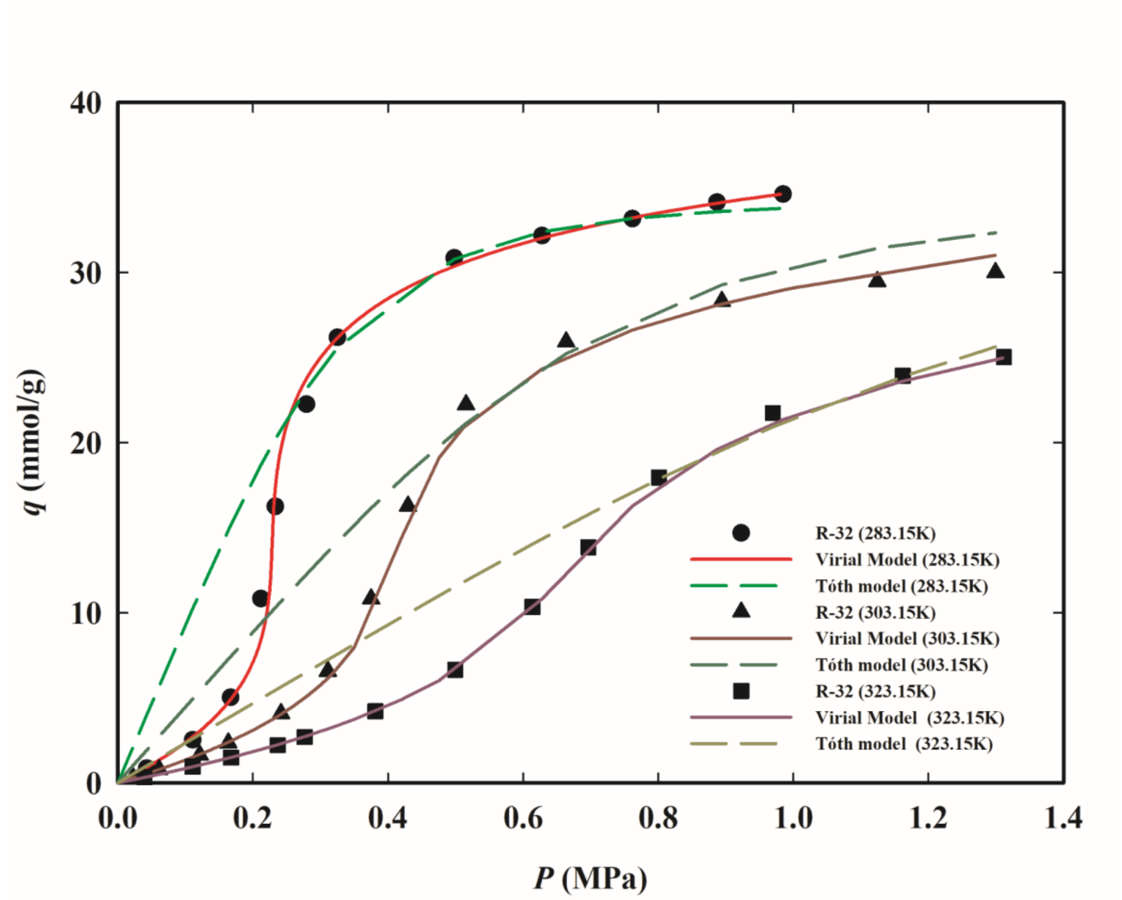

Figure S6. Comparison of the Tóth's and Virial models fitting to the adsorption of R-32 in MOF-177.

Table S6. Best-fit parameter values of the Tóth model for each adsorbent/F-gas pair.

		b	***t***	**Error (%)**
**Cu-BTC**	**R-32**	55.29	0.851	6.7
	**R-125**	8747	0.353	1.2
	**R-134a**	3713	0.520	6.0
**ZIF-8**	**R-32**	4.274	1.66	5.3
	**R-125**	43.79	1.01	6.5
	**R-134a**	43.62	1.09	5.7
**MIL-53(Al)**	**R-32**	12.22	0.481	1.5
	**R-125**	2629	0.256	0.56
	**R-134a**	7348	0.267	1.3
**ZSM-5**	**R-32**	3079	0.340	0.73
	**R-125**	4465	0.240	3.4
	**R-134a**	2260	0.254	0.67

Table S7. Viral model parameters obtained for the system MOF-177 + F-gas.

	**R-32**	**R-134a**	**R-125**
**A_0 (Kg/mol)**	-0.065	0.34	0.46
**A_1 (Kg·K/mol)**	7.747	-110.5	-144.6
**B_0 ((Kg/mol)^2)**	0.010	-0.066	-0.11
**B_1 ((Kg·K/mol)^2)**	-3.106	17.01	30.87
**C_0 ((Kg/mol)^3)**	0.000	0.004	0.006
**C_1 ((Kg/mol)^3)**	0.059	-0.89	-1.6
**K_∞ (mol/(Kg·bar))**	1.28E-02	3.61E-03	5.82E-03
**-ΔH (KJ/mol)**	17.260	19.380	21.869
**Error (%)**	0.107	1.06	1.33

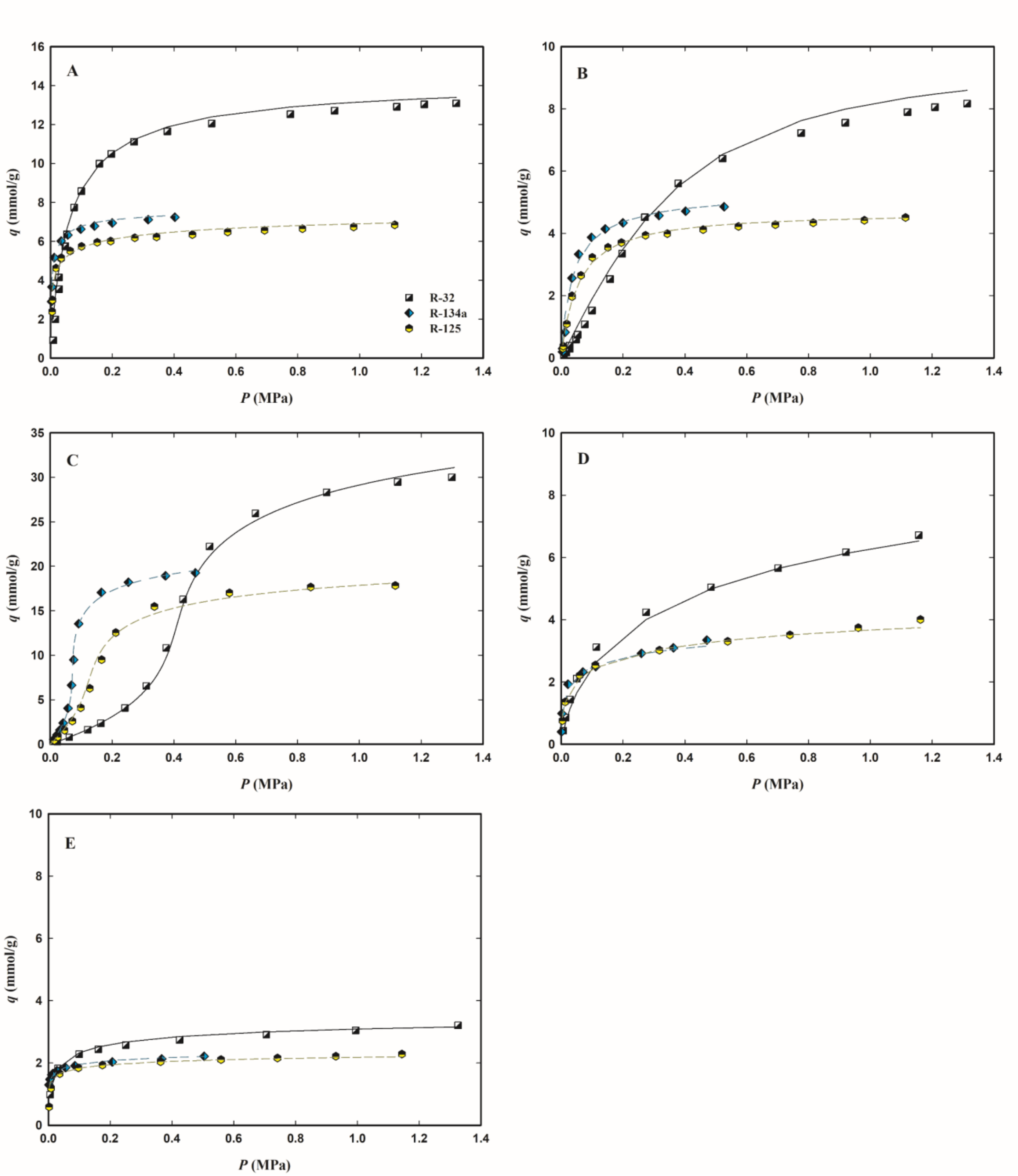

Figure S7. Adsorption equilibrium isotherms at 303.15 K of R-32 (squares), R-134a (diamond), and R-125 (hexagon) on Cu-BTC (panel A), ZIF-8 (panel B), MOF-177 (panel C), MIL-53(Al) (panel D), and ZSM-5 (panel E). The dashed lines represent the fittings with the Tóth's (panels A, B, D, and E) or the Virial models (panel C).

Table S8. Individual equilibrium loadings, *q* (mmol/g), obtained by IAST simulation for adsorption of R-410A ($y^{(0)}_{\text{R-32}} = 0.7$ and $y^{(0)}_{\text{R-125}} = 0.3$), R-32, and R-125 at 303.15 K. Pressure, *P*, is in MPa.

Cu-BTC			
P **(MPa)**	$q_{\text{R-410A}}$ **(mmol/g)**	$q_{\text{R-32}}$ **(mmol/g)**	$q_{\text{R-125}}$ **(mmol/g)**
0.01	3.42	0.601	2.82
0.03	4.78	1.33	3.45
0.05	5.53	1.90	3.63
0.08	6.31	2.62	3.69
0.10	6.71	3.04	3.67
0.15	7.50	3.96	3.54
0.20	8.11	4.75	3.36
0.25	8.61	5.44	3.17
0.30	9.05	6.06	2.98
0.40	9.75	7.14	2.62
0.50	10.3	8.04	2.28
0.60	10.8	8.81	1.98
0.70	11.2	9.47	1.71
0.80	11.5	10.0	1.48
0.90	11.8	10.5	1.27
1.00	12.0	10.9	1.09
**ZIF-8**			
P **(MPa)**	$q_{\text{R-410A}}$ **(mmol/g)**	$q_{\text{R-32}}$ **(mmol/g)**	$q_{\text{R-125}}$ **(mmol/g)**
0.01	0.378	0.135	0.243
0.03	1.02	0.383	0.642
0.05	1.56	0.606	0.952
0.08	2.20	0.902	1.30
0.10	2.55	1.08	1.48
0.15	3.25	1.46	1.79
0.20	3.77	1.79	1.98
0.25	4.17	2.07	2.10
0.30	4.50	2.32	2.17
0.40	5.00	2.75	2.25
0.50	5.37	3.11	2.26
0.60	5.67	3.42	2.25
0.70	5.91	3.69	2.22
0.80	6.11	3.94	2.17
0.90	6.29	4.16	2.13
1.00	6.44	4.36	2.08
**MOF-177**			
P **(MPa)**	$q_{\text{R-410A}}$ **(mmol/g)**	$q_{\text{R-32}}$ **(mmol/g)**	$q_{\text{R-125}}$ **(mmol/g)**
0.189	0.086	0.103	0.189
0.580	0.264	0.316	0.580
0.993	0.453	0.539	0.993

1.67	0.764	0.901	1.67
2.16	0.993	1.17	2.16
3.64	1.69	1.96	3.64
5.79	2.68	3.11	5.79
9.10	4.24	4.87	9.10
12.9	6.19	6.76	12.9
16.7	8.53	8.13	16.7
18.7	10.1	8.58	18.7
20.1	11.4	8.75	20.1
21.2	12.4	8.80	21.2
22.2	13.4	8.78	22.2
22.9	14.2	8.73	22.9
23.6	15.0	8.66	23.6
**MIL-53(Al)**			
***P* (MPa)**	**q_{R-410A} (mmol/g)**	**q_{R-32} (mmol/g)**	**q_{R-125} (mmol/g)**
0.01	0.923	0.193	0.731
0.03	1.50	0.429	1.07
0.05	1.84	0.612	1.23
0.08	2.21	0.842	1.37
0.10	2.40	0.976	1.43
0.15	2.79	1.27	1.52
0.20	3.09	1.52	1.57
0.25	3.34	1.75	1.59
0.30	3.55	1.95	1.60
0.40	3.92	2.31	1.60
0.50	4.22	2.63	1.59
0.60	4.48	2.92	1.56
0.70	4.70	3.17	1.53
0.80	4.91	3.41	1.50
0.90	5.09	3.63	1.46
1.00	5.26	3.83	1.42
**ZSM-5**			
***P* (MPa)**	**q_{R-410A} (mmol/g)**	**q_{R-32} (mmol/g)**	**q_{R-125} (mmol/g)**
0.01	1.33	0.294	1.04
0.03	1.64	0.553	1.09
0.05	1.80	0.733	1.07
0.08	1.96	0.943	1.02
0.10	2.04	1.06	0.983
0.15	2.19	1.29	0.900
0.20	2.31	1.48	0.824
0.25	2.40	1.64	0.758
0.30	2.47	1.77	0.700
0.40	2.59	1.99	0.601
0.50	2.68	2.15	0.522
0.60	2.75	2.29	0.457
0.70	2.81	2.40	0.403

0.80	2.86	2.50	0.358
0.90	2.90	2.58	0.320
1.00	2.94	2.65	0.287

Table S9. Individual equilibrium loadings, q_i (mmol/g), obtained by IAST simulation for adsorption of R-407F ($y^{(0)}_{\text{R-32}} = 0.47$, $y^{(0)}_{\text{R-125}} = 0.21$, and $y^{(0)}_{\text{R-134a}} = 0.32$), R-32, R-125, and R-134a at 303.15 K. Pressure, *P*, is in MPa.

**Cu-BTC**				
***P*(MPa)**	**$q_{\text{R-407F}}$(mmol/g)**	**$q_{\text{R-32}}$(mmol/g)**	**$q_{\text{R-125}}$(mmol/g)**	**$q_{\text{R-134a}}$(mmol/g)**
0.01	4.12	0.381	1.49	2.25
0.03	5.46	0.770	1.56	3.13
0.05	6.07	1.05	1.52	3.49
0.10	6.87	1.57	1.40	3.90
0.15	7.35	1.98	1.30	4.07
0.20	7.68	2.33	1.21	4.14
0.25	7.95	2.64	1.14	4.17
0.30	8.18	2.93	1.07	4.17
0.35	8.38	3.20	1.02	4.16
0.40	8.55	3.46	0.964	4.13
0.45	8.71	3.70	0.918	4.09
0.50	8.86	3.93	0.875	4.05
0.55	8.99	4.15	0.836	4.00
0.60	9.12	4.37	0.800	3.95
**ZIF-8**				
***P*(MPa)**	**$q_{\text{R-407F}}$(mmol/g)**	**$q_{\text{R-32}}$(mmol/g)**	**$q_{\text{R-125}}$(mmol/g)**	**$q_{\text{R-134a}}$(mmol/g)**
0.01	0.686	0.086	0.155	0.445
0.03	1.62	0.228	0.366	1.03
0.05	2.25	0.345	0.504	1.40
0.10	3.21	0.575	0.697	1.94
0.15	3.77	0.754	0.795	2.23
0.20	4.15	0.901	0.849	2.40
0.25	4.42	1.03	0.881	2.51
0.30	4.63	1.14	0.900	2.59
0.35	4.80	1.24	0.911	2.65
0.40	4.94	1.34	0.917	2.69
0.45	5.06	1.42	0.919	2.72
0.50	5.17	1.50	0.919	2.74
0.55	5.26	1.58	0.917	2.76
0.60	5.34	1.65	0.914	2.77
**MOF-177**				
***P*(MPa)**	**$q_{\text{R-407F}}$(mmol/g)**	**$q_{\text{R-32}}$(mmol/g)**	**$q_{\text{R-125}}$(mmol/g)**	**$q_{\text{R-134a}}$(mmol/g)**
0.01	0.283	0.058	0.073	0.152
0.03	0.880	0.181	0.225	0.474
0.05	1.54	0.318	0.392	0.830
0.10	3.81	0.787	0.954	2.07
0.15	10.3	2.00	2.40	5.95
0.20	14.5	2.88	3.14	8.50
0.25	16.2	3.41	3.34	9.47

0.30	17.3	3.83	3.43	10.0
0.35	18.1	4.20	3.48	10.4
0.40	18.7	4.52	3.51	10.7
0.45	19.2	4.81	3.52	10.9
0.50	19.7	5.09	3.53	11.1
0.55	20.1	5.34	3.53	11.2
0.60	20.4	5.58	3.54	11.3
		**MIL-53(Al)**		
***P*(MPa)**	**$q_{R\text{-}407F}$(mmol/g)**	**$q_{R\text{-}32}$(mmol/g)**	**$q_{R\text{-}125}$(mmol/g)**	**$q_{R\text{-}134a}$(mmol/g)**
0.01	1.18	0.098	0.292	0.794
0.03	1.72	0.211	0.419	1.09
0.05	2.01	0.300	0.485	1.23
0.10	2.45	0.477	0.577	1.39
0.15	2.73	0.625	0.631	1.48
0.20	2.95	0.755	0.668	1.52
0.25	3.12	0.874	0.696	1.55
0.30	3.27	0.984	0.718	1.57
0.35	3.40	1.09	0.736	1.58
0.40	3.52	1.19	0.751	1.58
0.45	3.63	1.28	0.763	1.59
0.50	3.73	1.37	0.773	1.58
0.55	3.82	1.46	0.782	1.58
0.60	3.91	1.55	0.789	1.58
		**ZSM-5**		
***P*(MPa)**	**$q_{R\text{-}407F}$(mmol/g)**	**$q_{R\text{-}32}$(mmol/g)**	**$q_{R\text{-}125}$(mmol/g)**	**$q_{R\text{-}134a}$(mmol/g)**
0.01	1.41	0.179	0.581	0.649
0.03	1.69	0.322	0.561	0.806
0.05	1.82	0.419	0.537	0.868
0.10	2.01	0.596	0.488	0.929
0.15	2.13	0.730	0.451	0.946
0.20	2.21	0.840	0.422	0.947
0.25	2.28	0.937	0.398	0.941
0.30	2.33	1.02	0.377	0.931
0.35	2.38	1.10	0.359	0.918
0.40	2.42	1.17	0.342	0.904
0.45	2.46	1.24	0.327	0.889
0.50	2.49	1.30	0.314	0.874
0.55	2.52	1.36	0.302	0.859
0.60	2.55	1.41	0.290	0.843

Table S10. Selectivities for the separation of R-32/R-125 at 303.15 K, assuming the molar fractions of the gas phase at the equilibrium of the commercial refrigerant R-410A.

		R-32/R-125			
***P* (MPa)**	**Cu-BTC**	**ZIF-8**	**MOF-177**	**MIL-53(Al)**	**ZSM-5**
0.01	0.091	0.238	0.355	0.113	0.121
0.03	0.166	0.256	0.358	0.172	0.218
0.05	0.225	0.273	0.360	0.213	0.295
0.08	0.305	0.297	0.363	0.264	0.397
0.10	0.356	0.313	0.365	0.293	0.461
0.15	0.480	0.351	0.369	0.358	0.617
0.20	0.605	0.388	0.370	0.416	0.771
0.25	0.735	0.423	0.373	0.470	0.926
0.30	0.871	0.458	0.392	0.521	1.08
0.40	1.17	0.524	0.450	0.618	1.42
0.50	1.51	0.589	0.505	0.711	1.77
0.60	1.91	0.652	0.557	0.801	2.15
0.70	2.37	0.714	0.606	0.890	2.55
0.80	2.91	0.776	0.653	0.978	2.99
0.90	3.54	0.837	0.698	1.07	3.46
1.00	4.29	0.897	0.741	1.15	3.95

Table S11. Selectivities for the separations of R-32/R-125, R-134a/R-32, and R-134a/R-125 at 303.15 K, assuming the molar fractions of the gas phase at the equilibrium of the commercial refrigerant R-407F.

	**Cu-BTC**		
***P* (MPa)**	**R-125/ R-32**	**R-134a/R-32**	**R-134a/R-125**
0.01	9.05	8.71	0.962
0.03	4.67	5.97	1.28
0.05	3.35	4.90	1.46
0.10	2.06	3.65	1.77
0.15	1.51	3.02	2.00
0.20	1.20	2.62	2.18
0.25	0.993	2.32	2.34
0.30	0.844	2.09	2.48
0.35	0.732	1.91	2.61
0.40	0.644	1.76	2.73
0.45	0.573	1.63	2.84
0.50	0.514	1.52	2.95
0.55	0.464	1.42	3.05
0.60	0.422	1.33	3.15
	**ZIF-8**		
***P* (MPa)**	**R-125/ R-32**	**R-134a/R-32**	**R-134a/R-125**
0.01	4.18	7.63	1.83
0.03	3.71	6.64	1.79
0.05	3.37	5.98	1.78
0.10	2.80	4.96	1.77
0.15	2.43	4.34	1.79
0.20	2.17	3.91	1.80
0.25	1.98	3.59	1.82
0.30	1.82	3.34	1.84
0.35	1.69	3.13	1.85
0.40	1.58	2.96	1.87
0.45	1.49	2.81	1.89
0.50	1.41	2.68	1.90
0.55	1.34	2.57	1.92
0.60	1.28	2.47	1.93
	**MOF-177**		
***P* (MPa)**	**R-125/ R-32**	**R-134a/R-32**	**R-134a/R-125**
0.01	2.90	3.88	1.34
0.03	2.87	3.85	1.34
0.05	2.84	3.84	1.35
0.10	2.80	3.87	1.38
0.15	2.77	4.37	1.58
0.20	2.52	4.35	1.73
0.25	2.26	4.09	1.81
0.30	2.06	3.85	1.87

0.35	1.91	3.65	1.91
0.40	1.79	3.48	1.94
0.45	1.69	3.33	1.98
0.50	1.60	3.20	2.00
0.55	1.53	3.09	2.02
0.60	1.46	2.98	2.04
**MIL-53(Al)**			
***P* (MPa)**	**R-125/ R-32**	**R-134a/R-32**	**R-134a/R-125**
0.01	6.91	12.0	1.73
0.03	4.58	7.59	1.66
0.05	3.73	6.02	1.61
0.10	2.79	4.30	1.54
0.15	2.33	3.48	1.49
0.20	2.04	2.97	1.45
0.25	1.84	2.61	1.42
0.30	1.68	2.35	1.39
0.35	1.56	2.13	1.37
0.40	1.46	1.96	1.35
0.45	1.37	1.82	1.33
0.50	1.30	1.70	1.31
0.55	1.23	1.59	1.29
0.60	1.18	1.50	1.27
**ZSM-5**			
***P* (MPa)**	**R-125/ R-32**	**R-134a/R-32**	**R-134a/R-125**
0.01	7.48	5.32	0.712
0.03	4.03	3.69	0.916
0.05	2.95	3.05	1.03
0.10	1.89	2.29	1.21
0.15	1.43	1.91	1.34
0.20	1.16	1.66	1.43
0.25	0.980	1.48	1.51
0.30	0.850	1.34	1.57
0.35	0.752	1.23	1.63
0.40	0.674	1.13	1.68
0.45	0.610	1.06	1.73
0.50	0.557	0.989	1.78
0.55	0.512	0.930	1.82
0.60	0.474	0.878	1.85

REFERENCES

[S1] D. K. J. A. Wanigarathna, J. Gao, B. Liu, *Chem. - An Asian J.* **2018**, *13*, 977–981. DOI: 10.1002/asia.201800337.
